# Aberrantly Expressed lncRNAs in Primary Varicose Great Saphenous Veins

**DOI:** 10.1371/journal.pone.0086156

**Published:** 2014-01-31

**Authors:** Xiang Li, Xiao-Yan Jiang, Jin Ge, Jing Wang, Guo-Jun Chen, Liang Xu, Duan-Yang Xie, Tian-You Yuan, Da-Sheng Zhang, Hong Zhang, Yi-Han Chen

**Affiliations:** 1 Key Laboratory of Arrhythmia of the Ministry of Education of China, East Hospital, Tongji University School of Medicine, Shanghai, China; 2 Institute of Medical Genetics, Tongji University, Shanghai, China; 3 Department of Pathology and Pathophysiology, Tongji University School of Medicine, Shanghai, China; 4 Department of Vascular Surgery, East Hospital, Tongji University School of Medicine, Shanghai, China; University of Toronto, Canada

## Abstract

Long non-coding RNAs (lncRNAs) are key regulatory molecules involved in a variety of biological processes and human diseases. However, the pathological effects of lncRNAs on primary varicose great saphenous veins (GSVs) remain unclear. The purpose of the present study was to identify aberrantly expressed lncRNAs involved in the prevalence of GSV varicosities and predict their potential functions. Using microarray with 33,045 lncRNA and 30,215 mRNA probes, 557 lncRNAs and 980 mRNAs that differed significantly in expression between the varicose great saphenous veins and control veins were identified in six pairs of samples. These lncRNAs were sub-grouped and mRNAs expressed at different levels were clustered into several pathways with six focused on metabolic pathways. Quantitative real-time PCR replication of nine lncRNAs was performed in 32 subjects, validating six lncRNAs (AF119885, AK021444, NR_027830, G36810, NR_027927, uc.345-). A coding-non-coding gene co-expression network revealed that four of these six lncRNAs may be correlated with 11 mRNAs and pathway analysis revealed that they may be correlated with another 8 mRNAs associated with metabolic pathways. In conclusion, aberrantly expressed lncRNAs for GSV varicosities were here systematically screened and validated and their functions were predicted. These findings provide novel insight into the physiology of lncRNAs and the pathogenesis of varicose veins for further investigation. These aberrantly expressed lncRNAs may serve as new therapeutic targets for varicose veins. The Human Ethnics Committee of Shanghai East Hospital, Tongji University School of Medicine approved the study (NO.: 2011-DF-53).

## Introduction

Varicose veins, whose manifestations can vary from leg edema to chronic, disabling venous ulceration, affect around 25% of the adult population and can lead to considerable morbidity and congestion of health service resources [1]. Great saphenous veins (GSVs) or saphenofemoral junction accounts for about 70% of varicose veins [1]–[3]. Many risk factors, such as increased age, female gender, diet rich in processed foods, obesity, work requiring large amounts of standing, tight undergarments, cigarette smoking, hypertension and diabetes mellitus, contribute to the prevalence of GSV varicosity [1], [3]. However, the molecular mechanisms underlying the connection between these factors that lead to the prevalence of varicose veins remain obscure.

Recent progress addressing the molecular mechanism of GSV varicosities includes venous valvular dysfunction-causing reflux [4], leukocyte diapedesis and local inflammation, smooth muscle cell apoptosis and proliferation, endothelial cell injury, increased vein wall tension, vein wall dilation, extracellular matrix degradation and tissue remodeling [3], [5], [6]. Many molecules from many different pathways are involved in the pathological processes associated with varicose veins. These molecules include hypoxia-inducible factor 1-alpha (HIF-1alpha) in the hypoxia pathway [7], the Janus kinase/signal transducers and activators of transcription (JAK-STAT) and nuclear factor kappaB (NF-kappaB) in the inflammatory pathway [8], poly ADP ribose polymerase (PARP) and bax in the apoptotic pathway [9], adhesion molecules and cytokines such as intercellular adhesion molecule 1 (ICAM-1), interleukin-1 alpha (IL-1alpha), and tumor necrosis factor alpha (TNF-alpha) [10]. However, these molecules account for only some of the tangled mechanisms, and many more remain to be identified. In addition, molecules such as long non-coding RNAs (lncRNAs) and micro-RNAs whose concentrations are correlated with those of their targets at the mRNA level, post-transcriptional level, or protein level must be systematically screened and validated.

LncRNAs are a class of transcripts whose lengths exceed 200 nt [11]. They are found throughout the genome. LncRNAs play key regulatory roles in regulating transcription in both cis form and antisense form, localization of proteins, and organizational frameworks of sub-cellular structures. LncRNAs also post-transcriptionally control mRNAs by affecting splicing, editing, translation, and degradation. In addition, many lncRNAs are processed into small RNAs or, modulate how other RNAs are processed. It is becoming increasingly clear that lncRNAs function in several different ways and play key roles in many intracellular regulatory processes [12], [13]. The abnormal regulation of lncRNAs is involved in several human diseases, such as cancer [14]–[16], Alzheimer’s disease [17], spinocerebellar ataxia (SCA) [18] and cardiovascular disease [19]–[25]. However, the relationship between lncRNAs and varicose veins is still unclear.

The present study was designed for screening and validation of lncRNAs at the micro-array level and examination of their relationship with the prevalence of varicose veins mRNAs and mRNA pathways associated with the aberrantly expressed lncRNAs, were identified at the whole micro-array level. These mRNAs and pathways may be relevant to the incidence of GSV varicosity.

## Materials and Methods

### Patients and Tissue Samples

Thirty-two samples of human primary GSVs were retrieved from 32 patients (14 men, 18 women) who were undergoing GSVs varicose vein excision in Shanghai East Hospital, Tongji University School of Medicine, China. The diagnosis of primary varicose GSVs was based on the clinical signs and duplex ultrasound scanning. All patients were characterized as having primary varicosities, and patients with secondary varicosities were excluded. None of the participants had any history of deep venous thrombosis, superficial thrombotic phlebitis, post-thrombotic syndrome, Klippel-Trenaunay syndrome, May-Thurner syndrome or any other venous disease. The clinical, etiological, anatomical and pathological elements classification system (CEAP) was used to classify chronic lower-extremity venous disease (CVD) in this case [26], [27]. The patients’ clinical signs placed all of them in classes 4–6, 30 of them were in class 4. Preoperative lower-extremity venous duplex ultrasound scanning assessment was performed on all patients, and examinations of both the superficial and the deep venous systems examination were conducted. All patients exhibited reflux in the GSVs. Patient demographics and clinical risk factors are given in **Table 1**.

**Table 1:** The clinical information of 32 patients involved in the study.

		Male	Female	Total	
**Patients number of gender**		14	18	32	
**Age±SD(years)**		54.4±13.2	54.9±8.9	54.7±10.8	
**Course of CVI(years)**		11.1±9.4	12.3±8.6	11.6±8.9	
**Previous chronic illnesses**	Hypertension	5	3	8	
	Parkinson	1	0	1	
	Diabetes	1	0	1	
**Limbs of Surgery**	left limb	5	5	10	
	right limb	7	8	15	
	double limbs	2	5	7	
**CEAP grade**	Class 6	1	0	1	
	Class 5	1	0	1	
	Class 4	12	18	30	
**Specialist physic examination**	Perthes test (−)	14	18	32	
**Duplex ultrasound scanning**	Deep venous thrombosis	0	2	2	
	Valve insufficiency of GSV	14	18	32	
	Reflux of GSV	14	18	32	

Paired tissues were used to evaluate the differences in expression level between varicose veins (VVs) and adjacent normal segments of saphenous veins (NVs). According to visual inspection and pathological examination, VV was the obvious varicose vein, and NV was the adjacent normal vein. NV samples were collected about 3–4 cm from the VV area. The tissues were snap-frozen into liquid nitrogen immediately after resection for later RNA extraction. Six pairs of samples were taken from six patients from 32 patients were used for lncRNAs expression microarray and all 32 samples were used for quantitative real-time PCR (Q-RT PCR) validation. Written informed consent was obtained from all participants. The study was approved by the Human Ethnics Committee of Shanghai East Hospital, Tongji University School of Medicine (NO.: 2011-DF-53).

### RNA Extraction and RNA Quantity Control

Total RNA was extracted from 32 pairs of samples which had been snap frozen using TRIzol reagent (Invitrogen, Carlsbad, CA, U.S.) according to the manufacturer’s protocol. The amount and quality of RNA were measured using NanoDrop ND-1000 and RNA integrity was assessed by standard denaturing agarose gel electrophoresis.

### RNA Labeling and Microarray Hybridization

Sample labeling and microarray hybridization were performed using a modified version of the Agilent One-Color Microarray-Based Gene Expression Analysis protocol (Agilent Technology). Briefly, rRNA was removed from the total RNA sample, and then the mRNA was purified (mRNA-ONLY™ Eukaryotic mRNA Isolation Kit, Epicentre). Then, each sample was amplified and transcribed into fluorescent cRNA along the entire length of the transcripts without 3′ bias using a random priming method. The labeled cRNAs were purified using an RNeasy Mini Kit (Qiagen). The concentration and specific activity of the labeled cRNAs (pmol Cy3/µg cRNA) were measured using NanoDrop ND-1000. Then 1 µg of each labeled cRNA was fragmented by adding 11 µl 10 × Blocking Agent and 2.2 µl of 25 × fragmentation buffer. Then the mixture was heated at 60°C for 30 min. Then 55 µl 2 × GE hybridization buffer was added to dilute the labeled cRNA. Then 100 µl of hybridization solution was dispensed into the gasket slide and placed in the Arraystar Human LncRNA Array v2.0 with 33,045 LncRNAs were collected from the authoritative data sources including RefSeq, UCSC Knowngenes, Ensembl and many related studies. The slides were incubated for 17 h at 65°C in an Agilent Hybridization Oven. The hybridized arrays were washed, fixed, and scanned with using the Agilent DNA Microarray Scanner (part number G2505B). The microarray work was performed by KangChen Bio-tech (Shanghai). The microarray data discussed in this article have been deposited in National Center for Biotechnology Information (NCBI) Gene Expression Omnibus (GEO) and are accessible through (GEO) Series accession number GSE51260 (http://www.ncbi.nlm.nih.gov/geo/query/acc.cgi?acc=GSE51260).

### Q-RT-PCR

Total RNA was extracted from frozen vein specimens using TRIzol reagent (Invitrogen Life Technologies) and then reverse transcribed using a PrimeScript ™ RT Reagent Kit (Takara) according to the manufacturer’s instructions. LncRNA expression in VV and paired NV tissues was measured by Q-RT-PCR using Power SYBR® Green PCR Master Mix (Applied Biosystems) on the ABI PRISM® 7900 Sequence Detection System (SDS) instrument. The Q-RT-PCR primers were designed using Primer 3.0 and blasted specifically in NCBI. Then 1 µg of total RNA was converted to cDNA according to the manufacturer’s protocol. PCR was performed in a total reaction volume of 10 µl, including 5 µl SYBR Green PCR Master Mix (2×), 0.4 µl each of PCR forward and reverse primer (10 µM), 0.5 µl of cDNA, and 3.7 µl of double-distilled water. The quantitative real-time PCR reaction was set at an initial denaturation step of 10 min at 95°C followed by 40 cycles of 95°C for 15 s and, 60°C for 1 min. All experiments were performed in triplicate.

### Data Computational Analysis

For lncRNAs and mRNA microarray screening analysis, Agilent Feature Extraction software (version 10.7.3.1) was used to analyze acquired array images. After quantile normalization of the raw data, lncRNAs and mRNAs were chosen for further data analysis. A volcano plot was used to filter lncRNAs/mRNAs that were differentially expressed between two groups and Hierarchical clustering was performed using the GeneSpring GX v12.0 software package (Agilent Technologies). LncRNAs that were significantly differently expressed between all six pairs of VV and NV tissues were selected for Q-RT-PCR validation (*P*<0.05, ≥2 fold-change), as were any neighbor mRNAs that were also significantly differently expressed between all six pairs of VV and NV tissues (*P*<0.05, ≥2 fold-change).

For Q-RT-PCR validation analysis, all samples were normalized to GAPDH. The mean value in each triplicate was used to calculate relative lncRNAs concentrations (ΔCt = Ct _mean lncRNAs_−Ct _mean GAPDH_). Expression fold changes were calculated using 2^−ΔΔCt^ methods. The differences in the level of lncRNAs expression between VVs and NVs were analyzed using the Student’s t-test and SPSS (Version 16.0, SPSS Inc.) with the value of *P*<0.05 was considered as statistically significant.

Gene Ontology (GO) analysis and pathway analysis were performed to identify differentially expressed mRNA pathways. GO analysis showed significantly up-regulated and down-regulated mRNAs in question to be related to biological processes (BP), cellular components (CC) and molecular functions (MF) (*P*<0.05) The latest version of the Kyoto Encyclopedia of Genes and Genomes (KEGG) database and GO categories derived from Gene Ontology (www.geneontology.org) were used for pathway analysis, which was performed using the standard enrichment computation method [28]. A coding-non-coding gene co-expression network (CNC network) was drawn using Cytoscape with Pearson coefficient (|r|) >0.99 [29], [30]. The analysis work was performed by KangChen Bio-tech (Shanghai P.R. China).

## Results

### Profiles of the Differently Expressed lncRNAs and mRNAs

Among the 33,045 lncRNAs and 30,215 coding transcripts probed in the microarray, 12,264 lncRNAs and 14,862 mRNAs were detected in all six pairs of samples. There was an average of 2,426 differentially expressed lncRNAs (**Table S1**) (ranging from 1,442 to 4,049 depending on the subjects) and 3,029 differentially expressed mRNAs (**Table S2**) (ranging from 1,914 to 5,063). Among them, 557 lncRNAs (302 up-regulated and 255 down-regulated) and 980 mRNAs (239 up-regulated and 741 down-regulated) were significantly differently expressed (≥2 fold) (**Table 2** **)**. Among the 20 most significantly differentially expressed lncRNAs **(** **Table 3** **)**, BC041954 (fold change≈ 23.1) was the most significantly up-regulated lncRNA and AK023929 was the most significantly down-regulated (fold change = 4.84)**.** There were more down-regulated lncRNAs than up-regulate lncRNAs.

**Table 2:** Numbers of LncRNA and mRNA expressed differently between six pairs of VVs and paired NVs tissues.

	Fold change2–4	Fold change4–8	Fold change >8	total
**LncRNA**				
Up-regulation	300	2	1	302
Down-regulation	249	6	0	255
**mRNA**				
Up-regulation	236	3	0	239
Down-regulation	708	32	1	741

**Table 3:** Top 20 significantly differential expressed LncRNAs from the microarray data.

LncRNA accession number	Regulation	Fold change	Source	FDR	SD
BC041954	up	23.1	misc_RNA	0.0184	0.0334
chr10∶127700955–127703336	up	4.25	lncRNAdb	0.0012	0.0194
AK097695	up	4.18	RNAdb	0.0019	0.0194
ENST00000424739	up	3.97	Ensembl	0.0020	0.0194
ENST00000484962	up	3.77	Ensembl	0.0089	0.0274
BC042185	up	3.77	RNAdb	0.0395	0.0474
ENST00000512300	up	3.62	Ensembl	0.0140	0.0299
ENST00000445497	up	3.57	Ensembl	0.0068	0.0247
ENST00000494923	up	3.47	Ensembl	0.0131	0.0290
ENST00000448961	up	3.33	Ensembl	0.0014	0.0194
ENST00000510464	up	3.19	Ensembl	0.0305	0.0423
ENST00000450430	up	3.18	Ensembl	0.0318	0.0423
nc-HOXB9-205-	up	3.16	HOX cluster	0.0068	0.0247
ENST00000437515	up	3.15	Ensembl	0.0082	0.0266
uc.459-	up	3.15	UCR	0.0438	0.0487
ENST00000392399	up	3.09	Ensembl	0.0100	0.0285
ENST00000428292	up	3.09	Ensembl	0.0163	0.0326
BC002831	up	3.09	misc_RNA	0.0069	0.0248
chr7∶117584264–117586960+	up	3.08	lincRNA	0.0082	0.0266
AM492791	up	3.03	misc_RNA	0.0346	0.0441
AK023929	down	4.84	NRED	0.0045	0.0218
AK126867	down	4.57	misc_RNA	0.0092	0.0278
uc002bmd.2	down	4.39	UCSC_knowngene	0.0039	0.0200
ENST00000427890	down	4.28	Ensembl	0.0223	0.0368
AK126761	down	4.11	misc_RNA	0.0462	0.0487
ENST00000429435	down	4.03	Ensembl	0.0124	0.0290
uc.28+	down	3.89	UCR	0.0029	0.0197
AK297077	down	3.83	lincRNA	0.0107	0.0286
BX648634	down	3.82	misc_RNA	0.0462	0.0487
NR_026570	down	3.79	RefSeq_NR	0.0060	0.0246
NR_015407	down	3.62	RefSeq_NR	0.0385	0.0466
AK022120	down	3.57	NRED	0.0093	0.0278
CR624187	down	3.57	RNAdb	0.0485	0.0494
ENST00000474978	down	3.53	Ensembl	0.0278	0.0410
NR_027830	down	3.48	RefSeq_NR	0.0005	0.0194
uc003jev.1	down	3.46	UCSC_knowngene	0.0107	0.0286
ENST00000420004	down	3.43	Ensembl	0.0041	0.0205
ENST00000423135	down	3.38	Ensembl	0.0025	0.0194
AK091713	down	3.37	NRED	0.0058	0.0246
ENST00000455804	down	3.34	Ensembl	0.0017	0.0194

Sourse: different database each lncRNA was included; LncRNA accession number: the reference ID of lncRNA in each database.

The GO analytical data of aberrantly expressed mRNAs are shown in **Table S3** (for biological processes), **Table S4** (for cellular components), and **Table S5** (for molecular functions). The top 20 most significantly differentially expressed mRNAs are shown in **Table 4**, and the top 5 most enriched GO terms, specifically biological processes, cellular components and molecular functions, are shown in **Table 5**. Pathway analysis showed these 123 significantly differentially expressed mRNAs to be involved in the pathogenesis of GSV varicosities **(Table S6)**. Among them, 26 down-regulated and 2 up-regulated pathways were detected and 7 pathways were found to contain 46 mRNAs (changed ≥2 fold, enrichment score (−log10 (*P* -value)) value >2, *P*<0.01). Among these pathways, six (glycolysis/gluconeogenesis, fatty acid metabolism, tyrosine metabolism, pyrimidine metabolism, peroxisome and maturity-onset diabetes of the young) were focused on metabolic pathways.

**Table 4:** Top 20 significantly differential expressed mRNAs from the microarray data.

mRNA genesymbol	NCBIaccession	Regulation	Foldchange	FDR	SD
POSTN	NM_001135936	up	5.99	9.50E-03	1.92
PRND	NM_012409	up	4.85	9.20E-03	1.84
POSTN	NM_001135935	up	4.41	8.30E-03	1.77
PPARGC1B	NM_133263	up	4.05	4.55E-02	1.71
FAM64A	NM_019013	up	3.77	2.34E-02	1.41
PODXL	NM_001018111	up	3.49	1.04E-02	1.37
ALPL	NM_000478	up	3.47	1.02E-02	1.18
PRSS2	NM_002770	up	3.46	1.24E-02	1.59
KRT17	NM_000422	up	3.33	8.30E-03	1.42
POSTN	NM_001135934	up	3.31	7.70E-03	1.22
ADAMTS14	NM_080722	up	3.31	2.13E-02	1.50
C9orf140	NM_178448	up	3.28	2.88E-02	1.58
PCSK9	NM_174936	up	3.25	7.70E-03	1.23
RBP4	NM_006744	up	3.17	7.70E-03	1.08
LRRC15	NM_001135057	up	3.16	2.48E-02	1.48
GINS2	NM_016095	up	3.09	1.96E-02	1.45
TK1	NM_003258	up	3.08	1.18E-02	1.12
MAGEL2	NM_019066	up	3.08	7.70E-03	1.20
NECAB2	NM_019065	up	3.04	9.00E-03	1.06
SLC22A14	NM_004803	up	3.03	2.56E-02	1.27
MYOC	NM_000261	down	8.46	1.08E-02	1.92
CUL2	NM_003591	down	6.81	1.27E-02	1.81
CNN3	NM_001839	down	6.52	1.37E-02	2.65
7-Sep	NM_001011553	down	6.19	7.70E-03	1.69
SRP54	NM_003136	down	5.89	7.70E-03	1.54
ADH1A	NM_000667	down	5.87	8.30E-03	1.46
ADH1B	NM_000668	down	5.62	1.08E-02	1.50
KDM3B	NM_016604	down	5.55	9.50E-03	1.58
IDH1	NM_005896	down	5.08	7.70E-03	1.51
HRNBP3	NM_001082575	down	5.08	1.67E-02	2.35
DHX40	NM_024612	down	5.00	7.70E-03	1.33
MIS12	NM_024039	down	4.85	8.30E-03	1.32
CXCL1	NM_001511	down	4.74	8.40E-03	1.54
SEC63	NM_007214	down	4.67	7.70E-03	1.45
RNF103	NM_005667	down	4.67	9.90E-03	1.52
S100A8	NM_002964	down	4.57	1.95E-02	1.79
UEVLD	NM_001040697	down	4.56	1.07E-02	1.46
PTPRK	NM_001135648	down	4.53	7.70E-03	1.41
PDK4	NM_002612	down	4.40	8.40E-03	1.31
MAPRE2	NM_014268	down	4.30	8.50E-03	1.40

NCBI accession: the reference ID of mRNA in NCBI (National Center for Biotechnology Information).

**Table 5:** Top 5 Enrichment GO term (BP, CC and MF) from the microarray data.

GO.ID	Term	Ontology	Regulation	Enrichment.Score	FDR
GO:0044237	cellular metabolic process	BP	down	6.96	2.47E-04
GO:0006996	organelle organization	BP	down	6.75	2.47E-04
GO:0044238	primary metabolic process	BP	down	6.69	2.47E-04
GO:0008152	metabolic process	BP	down	5.72	1.76E-03
GO:0044260	cellular macromolecule metabolic process	BP	down	5.52	2.19E-03
GO:0044424	intracellular part	CC	down	23.63	1.21E-21
GO:0005622	intracellular	CC	down	22.97	2.77E-21
GO:0043229	intracellular organelle	CC	down	16.24	8.68E-15
GO:0043231	intracellular membrane-bounded organelle	CC	down	16.11	8.68E-15
GO:0043226	organelle	CC	down	16.06	8.68E-15
GO:0005488	binding	MF	down	9.43	3.30E-07
GO:0005515	protein binding	MF	down	6.37	1.87E-04
GO:0003824	catalytic activity	MF	down	5.59	7.50E-04
GO:0003723	RNA binding	MF	down	4.94	2.51E-03
GO:0000166	nucleotide binding	MF	down	4.53	5.26E-03

FDR<0.05.

### Differently Expressed lncRNA Subgroups

Overall, 48 enhancer-like lncRNAs were found to be differentially expressed **(Table S7)** using with Gencode annotation [31], [32]. Their nearby significantly differentially expressed coding RNAs (distance<300 kb) are shown in **Table S8.** The Arraystar Human LncRNA Array v2.0 microarray contains profiling data of all probes in the 4 HOX loci, targeting 407 discrete transcribed regions, lncRNAs and coding transcripts. Among them, 10 lncRNAs and 10 coding transcripts were found to be differentially expressed in the human HOX loci of VVs and those of paired NVs **(Table S9).** Seventy-eight Rinn’s lincRNAs **(Table S10),** 14 lincRNAs, and 19 nearby coding RNAs found to be significantly differentially expressed **(Table S11)** [33], [34].

### Validation of Aberrantly Expressed Candidate lncRNAs

**Figure 1:**
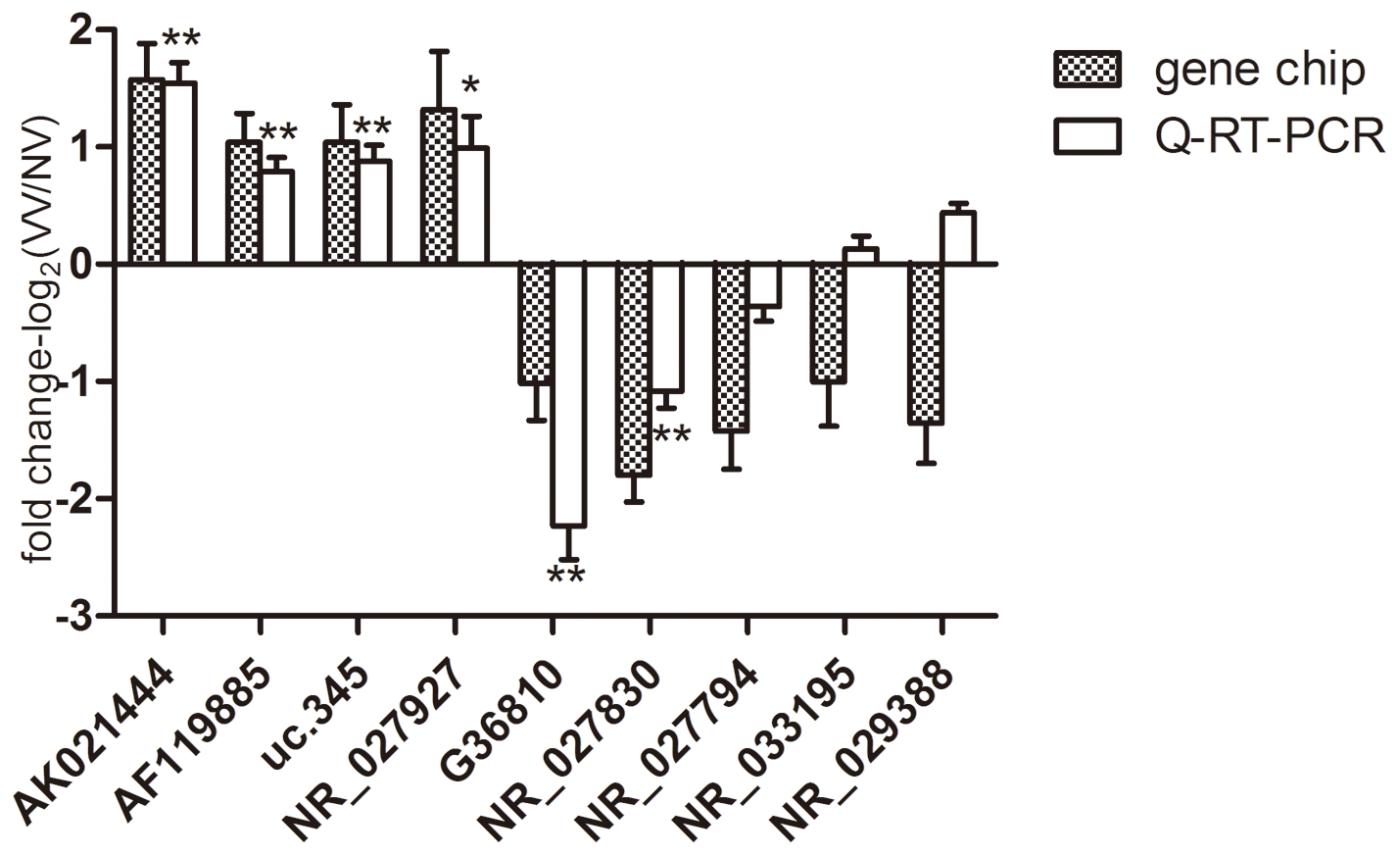
Validation of microarray data and the Q-RT-PCR data. Nine lncRNAs were chosen for validation in 32 pairs of VVs samples compared with NVs samples by Q-RT-PCR. seven of the nine lncRNAs showed the same trends with respect to up- or down- regulation as the microarray data and six lncRNAs (AK021444, AF119885, G36810, uc.345, NR_027927 and NR_027830) showed statistically significant differences (*P*<0.05). The heights of the columns in the chart represent the mean expression value of log2 fold changes (VVs/NVs) for each of the nine validated lncRNAs in the microarray and Q-RT-PCR data; The bars represent standard errors. The validation results indicated that the microarray data were closely correlate with the Q-RT-PCR results. *: *P*<0.05, **: *P*<0.01.

LncRNAs significantly differently expressed between all six pairs of VV and NV tissues with their neighbor mRNAs significantly differently expressed between all six pairs of VV and NV tissues were selected for Q-RT-PCR validation, and 22 lncRNAs were validated. Nine of the 22 lncRNAs whose Q-RT-PCR primers were suitably designed (using Primer 3.0 and blasted specifically in NCBI). Primers are shown in **Table S12.** These lncRNAs were then replicated with Q-RT-PCR. Of these nine lncRNAs, seven showed the same trends of up- and down- regulation as the microarray data and six were statistically significant (*P*<0.05) (**Figure 1** **)**, supporting a strong consistency between the Q-RT-PCR results and the microarray data.

### Prediction of the Functions of the Validated lncRNAs

**Figure 2:**
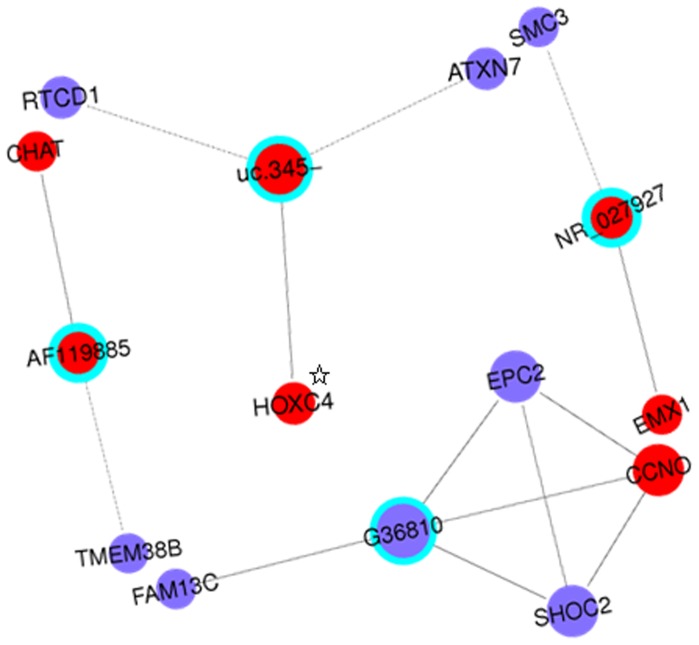
Coding-non-coding gene co-expression network of the four lncRNAs. The network represents co-expression correlations between the four lncRNAs and significantly differentially expressed mRNAs. Only co-expression gene pairs with Pearson coefficient (|r|)>0.99 are shown. Four separate networks were constructed. They are displayed together in this figure. Gene nodes with a cyan node lines represent lncRNAs and gene nodes without node lines represents a protein-coding RNA (mRNA). Red nodes represent up-regulated RNAs, and blue nodes represent down-regulated RNAs. Solid lines between two nodes indicate positively correlated interactions between RNAs, and dotted lines indicate negatively correlated interactions. Node size represents the node degrees. ☆indicates protein coding RNA transcribed from natural antisense strands of the gene HOXC4.

To study the relationship between the lncRNAs and mRNAs more visually, the validated six significantly differently expressed lncRNAs were used to establish coding-non-coding gene (CNC) networks. These CNC networks were used to search for correlations between the lncRNAs and mRNAs and to determine the potential function of the lncRNAs. This would increase understanding of lncRNAs biological networks and of the complex pathogenesis of GSV varicosities. Overall 11 significantly aberrantly expressed mRNAs were found to be correlated with four validated lncRNAs. They were used to construct four separate networks with lncRNAs in the center node of the hub. The mRNAs CHAT and TMEM38B were found to be related to lncRNA AF119885; the mRNAs CCNO, EPC2, FAM13C and SHOC2 were found to be related to lncRNA G36810; the mRNAs EMX1 and SMC3 were found to be related to lncRNA NR_027927; and the mRNAs ATXN7, HOXC4,and RTCD1 were found to be related to lncRNA uc.345-. These separate networks are shown in Figure 2 as a whole. Further information (standard deviation (SD), and false discovery rate (FDR)) regarding these 11 mRNAs is shown in **Table 6**.

**Table 6:** The 11 significantly aberrantly expressed mRNAs correlated with the four validated lncRNAs.

mRNAgenesymbol	NCBIaccession	Regulation	Foldchange	FDR	SD
CCNO	NM_021147	up	2.48	0.0102	1.084
CHAT	NM_020986	up	2.27	0.0090	0.792
HOXC4	NM_014620	up	2.02	0.0152	0.807
EMX1	NM_004097	up	2.01	0.0223	0.876
SHOC2	NM_007373	down	3.04	0.0083	1.144
TMEM38B	NM_018112	down	2.51	0.0077	0.854
SMC3	NM_005445	down	2.46	0.0086	0.850
EPC2	NM_015630	down	2.37	0.0077	0.817
FAM13C	NM_198215	down	2.32	0.0077	0.749
RTCD1	NM_003729	down	2.21	0.0155	0.788
ATXN7	NM_000333	down	2.02	0.0184	0.755

NCBI accession: the standard reference ID of mRNA in NCBI (National Center for Biotechnology Information).

The potential functional effects of the six validated candidate lncRNAs were predicted through their correlations with the aforementioned six GSV-varicosities -related mRNA metabolic pathways (glycolysis/gluconeogenesis, fatty acid metabolism, tyrosine metabolism, pyrimidine metabolism, peroxisome and maturity onset diabetes of the young) with a criteria of co-expression gene pairs with |r|>0.9 and P<0.01. Here, eight mRNAs were found to be correlated with specific lncRNAs (uc.345 with NME7; G36810 with POLR3F, IDH1, and ALDH2; AF119885 with ENTPD1, SLC25A17, and PDHA1; NR_027927 with NEUROG3). **Table 7** shows the mRNA pathways and pathway IDs.

**Table 7:** Interactional lncRNAs-mRNAs detected from significantly different expressed lncRNAs and mRNAs.

LncRNA	LncRNA fold change	Related mRNA	mRNA fold change	Involved pathway	Pathway ID
uc.345-	2.06	NME7	−2.60	Pyrimidine metabolism	hsa00240
G36810	−2.02	POLR3F	−4.18	Pyrimidine metabolism	hsa00240
		IDH1	−5.08	Peroxisome	hsa04146
		ALDH2	−2.31	Fatty acid metabolism	hsa00071
AF119885	2.05	ENTPD1	−2.34	Pyrimidine metabolism	hsa00240
		SLC25A17	−2.09	Peroxisome	hsa04146
		PDHA1	−2.08	Glycolysis/Gluconeogenesis	hsa00010
NR_027927	2.49	NEUROG3	2.79	MODY	hsa04950

coefficient (|r|) >0.9; FDR <0.01; *P*<0.01.

## Discussion

In the present study, the profiles of aberrantly expressed lncRNAs, mRNAs, and lncRNA-related mRNAs and pathways in primary varicose GSVs were evaluated. Six potential pathogenic lncRNAs were identified by Q-RT-PCR. The potential functional effects of these lncRNAs were predicted. This is the first systemic screening and validation of the GSV varicosities related lncRNAs in the genome-wide RNAs expression level.

The aberrantly expressed lncRNA patterns were classified into different subgroups with different analytical methods. Forty-eight enhancer-like lncRNAs were found to be differently expressed using GENCODE annotation, 10 discrete transcribed regions were targeted by 4 HOX loci, and 78 Rinn’s lincRNAs were identified using chromatin-state maps. Enhancer-like lncRNAs were associated with decreased expression of their neighboring genes through loss-of-function effects [31]. A total of 407 discrete transcribed regions including exons (101), introns (75), and intergenic transcripts (231) were identified in the 4 HOX loci [35]. Rinn’s lincRNAs profiles showed strong purifying selection, clear evolutionary conservation, and possible functions in many different biological processes [33], [34]. The aberrantly expressed lncRNAs observed may provide clues to the pathophysiological properties of GSV varicosities.

Six validated candidate lncRNAs (AK021444, AF119885, uc.345-, NR_027927, G36810, NR_027830) were identified in GSV varicosities by Q-RT-PCR and their potential functional effects were predicted. AK021444 is a 1611 bp lncRNA with an exon sense-overlapping relationship with POSTN. POSTN encods the protein periostin, which promotes cardiac repair and cardiomyocyte proliferation [36] and induces vascular cell differentiation and migration during the repair of vascular injury [37]. AF119885 is a 1269 bp lncRNA transcribed from the natural antisense strand of the gene ABCA10. The membrane-associated protein encoded by ABCA10 transports various molecules across extra and intracellular membranes. Uc.345 is a 301 bp lncRNA transcribed from the natural antisense strand of the gene HOXC4. HOXC4 belongs to the homeobox family of genes, which encode a highly conserved family of transcription factors that play an important role in morphogenesis in all multicellular organisms. NR_027927 is a 2249 bp lncRNA with a bidirectional relationship with the gene CAPN7. CAPN7 protein is an intracellular, nonlysosomal cysteine protease whose activity is regulated by calcium influx and oxidative stress [38]. G36810 is a 401 bp lncRNA transcribed from the natural antisense strand of gene TMEM47. This gene encodes a member of the PMP22/EMP/claudin protein family, which is localized to the ER and the plasma membrane. NR_027830 is a 5928 bp lncRNA with an exon sense-overlapping relationship with the gene BCAP29. BCAP29 may be involved in the anterograde transport of membrane proteins from the endoplasmic reticulum to the Golgi and in CASP8-mediated apoptosis. These data suggest that the six candidate lncRNAs may function in the etiology and pathogenesis of primary varicose GSVs, but further study is required to confirm this.

In this study, bio-informatics strategy was used to show that many lncRNAs expressions are correlated with that of nearby mRNAs expressions **(Table S13).** This is consistent with one proposed mechanism that lncRNAs are associated with related mRNAs [12]. Four of the six validated lncRNAs were associated with 11 significantly differentially expressed mRNAs. These mRNAs were involved in various biological processes. The biological functions of these genes which transcribed the 11 mRNAs are given in **Table S14.** These genes are involved in pathways involving the cell cycle (CCNO, SMC3), DNA repair (EPC2), RNA metabolism (RTCD1), neurological function (CHAT, FAM13C, EMX1, ATXN7, and HOXC4) and intracellular signaling (TMEM38B, SHOC2). However, the mechanisms by which they are connected to the designated lncRNAs and so to the prevalence of GSVs remain unknown. The lncRNAs and related gene pathways detected in our study suggest the complicated molecular mechanism of varicose veins.

**Figure 3:**
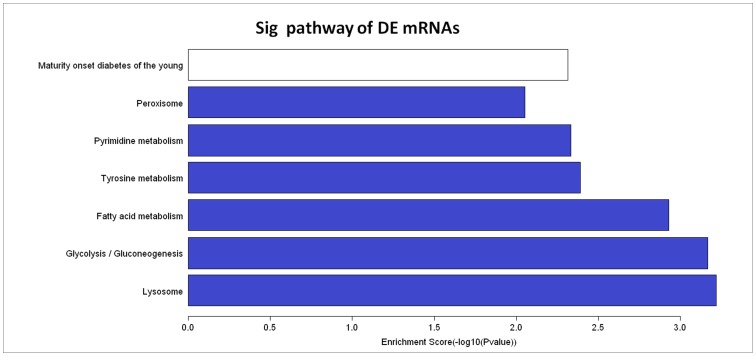
Pathways of dysregulated mRNAs with the enrichment scores (−log10 (*P*-value))>2. The bar plot shows the enrichment scores (−log10 (*P*-value)) value of the significant enrichment pathways. The white bar shows the pathway in which the up-regulated mRNAs were found to be involved and the blue bars show the pathways in which the down-regulated mRNAs were found to be involved. Pathway analysis involves mapping genes to KEGG pathways. The *P*-value denotes the significance of the correlation between the pathway and the conditions. Most of the shown here are related to metabolism, which indicates that the varicose veins may be a metabolic disease.

The significantly aberrantly expressed mRNAs were clustered into 7 pathways that were involved in the pathophysiological properties of GSV varicosity **(** **Figure 3** **)**. Of these, six are metabolic pathways. These pathways are involved in lysosomes, peroxisomes, glycolysis, diabetes, and tyrosine metabolism. Lysosomes transport the iron to the cytoplasmic labile iron pool [39] and glycolysis is a central molecule in glucose metabolism [40]. Peroxisomes participate in many crucial metabolic processes such as fatty acid oxidation, biosynthesis of ether lipids and free radical detoxification [41]. Maturity onset diabetes of the young (MODY) is an autosomal dominant form of diabetes involving defective β cells. Another three pathways, fatty acid metabolism, tyrosine metabolism and pyrimidine metabolism, are related to the amino acid metabolism. Taken together, these associations suggest that metabolic processes are involved in the pathogenesis of GSV varicosity.

The strength of this study should be addressed. Paired tissues taken from GSV varicosity patients were used to evaluate differences in expression between tissues from lesions and from adjacent normal segments of the saphenous vein. Unlike studies in which saphenous veins from healthy subjects were served as controls, this study was not subject to confounders associated with differences in genetic backgrounds, environmental exposure, or lifestyle. In addition, subjects with symptoms of valve insufficiency and reflux of GSVs were the focus of the present work. This diminished the heterogeneity of the studied phenotype, and so reduced the likelihood of false positives. The criteria used to select lncRNA for Q-RT-PCR validation was very robust. The limitations of this study should also be addressed. First, due to the lack of experimental validation, the bio-computationally derived links between lncRNAs and either individual mRNA or mRNA pathways should be viewed as preliminary. Second, varicose veins typically contain inflamed tissue, and inflammation is an important mechanism for the development of varicose veins. In addition, changes in mRNA and lncRNA levels may reflect pathological changes of inflamed tissue [42], [43]. However, since we did not strip the NVs of the additional lymphatic tissue and connective tissue that can build up in these samples, we could not distinguish whether or not the enriched lncRNAs and mRNAs are attributed to the additional cell types that are naturally more prevalent in inflamed tissue, which call for further study.

In summary, we systematically screened, validated, and functional predicted the aberrantly expressed lncRNAs primary varicose GSVs. These findings provide novel insights into physiology of lncRNAs and pathophysiological properties of GSV varicosities. These data may be useful in further investigation. The aberrantly expressed lncRNAs may also serve as new therapeutic targets for varicose veins.

## Supporting Information

Table S1 **Differentially Expressed LncRNAs.**
(XLS)

Table S2 **Differentially Expressed mRNAs.**
(XLS)

Table S3 **Biological Processes.**
(XLS)

Table S4 **Cellular Components.**
(XLS)

Table S5 **Molecular Functions.**
(XLS)

Table S6 **Pathway Analysis.**
(XLS)

Table S7 **Differentially Expressed Enhancer LncRNA.**
(XLS)

Table S8 **Enhancer LncRNAs nearby coding gene data.**
(XLS)

Table S9 **HOX cluster profiling.**
(XLS)

Table S10 **Differentially Expressed Rinn LincRNAs.**
(XLS)

Table S11 **LincRNAs and their nearby coding gene data table.**
(XLS)

Table S12 **Primers used for Q-RT-PCR.**
(XLS)

Table S13 **Differentially Expressed both LncRNAs and their related mRNAs.**
(XLS)

Table S14 **Function of the genes correlated the candidate lncRNAs.**
(XLS)
